# Crystal structure of naltrexone chloride solvates with ethanol, propan-2-ol, and 2-methyl­propan-2-ol

**DOI:** 10.1107/S205698901700843X

**Published:** 2017-06-13

**Authors:** Aveary R. Menze, Jefferson P. Sinnott, Alexander Y. Nazarenko

**Affiliations:** a Chemistry Department, SUNY Buffalo State, 1300 Elmwood Ave, Buffalo, NY 14222, USA

**Keywords:** crystal structure, naltrexone, chloride, solvate, ethanol, propan-2-ol, 2-methyl­propan-2-ol

## Abstract

Naltrexone [systematic name: 17-(cyclo­propyl­meth­yl)-3,14-di­hydroxy-4,5α-epoxymorphinan-6-one] is an opioid receptor competitive antagonist that has been widely used to prevent relapse in opioid- and alcohol-dependent subjects. Its chloride salt forms non-isomorphic solvates with ethanol (C_20_H_24_NO_4_^+^·Cl^−^·C_2_H_5_OH) (I), propan-2-ol (C_20_H_24_NO_4_^+^·Cl^−^·C_3_H_7_OH) (II), and 2-methyl­propan-2-ol (C_20_H_24_NO_4_^+^·Cl^−^·C_4_H_9_OH) (III). The naltrexone cation can be described as a T-shape made out of two ring systems, a tetra­hydro-2*H*-naphtho­[1,8-*bc*]furan system and a deca­hydro­isoquinolinium subunit, that are nearly perpendicular to one another. The flexible cyclo­propyl­methyl group can adopt various different conformations in response to its surroundings: an increase of available space around cyclo­propyl­methyl group may allow it to adopt a more favorable conformation. In all these structures, the alcohol mol­ecules occupy infinite solvent-filled channels. All three compounds described are attractive crystalline forms for unambiguous identification of naltrexone chloride after isolation from a pharmaceutical form. Compound (III) was refined as a two-component twin.

## Abstract 2

Naltrexone [systematic name: 17-(cyclo­propyl­meth­yl)-4,5α-ep­oxy-3,14-di­hydroxy­morphinan-6-one] is an opioid receptor competitive antagonist that has been widely used to prevent relapse in opioid- and alcohol-dependent subjects. Its chloride salt forms non-isomorphic solvates with ethanol (C_20_H_24_O_4_^+^·Cl^−^·C_2_H_5_OH) (I), propan-2-ol (C_20_H_24_O_4_^+^·Cl^−^·C_3_H_7_OH) (II), and 2-methyl­propan-2-ol (C_20_H_24_O_4_^+^·Cl^−^·C_4_H_9_OH) (III). In all these structures, the alcohol mol­ecules occupy infinite solvent-filled channels. All three compounds described are attractive crystalline forms for unambiguous identification of naltrexone chloride after isolation from a pharmaceutical form.

## Chemical context   

Alcohol and opiate dependence are potentially life-threatening disorders associated with adverse physical and societal effects including poor social functioning, familial problems, and crime (Compton & Volkow, 2006 ▸). One strategy suggested to address these issues is the inclusion of receptor antagonists that reduce, and can even reverse, the euphoric effects of the drug sought by abusers. Naltrexone [systematic name: 17-(cyclo­propyl­meth­yl)-3,14-di­hydroxy-4,5α-ep­oxy-morphinan-6-one] is an opioid receptor competitive antagonist that has been widely used to prevent relapse in heroin and other opioid-dependent subjects, and has been found to reduce cravings in alcohol-dependent subjects (Roozen *et al.*, 2006 ▸). Its structure-related analogue oxymorphone is a potent μ-agonist, which differs from naltrexone only in having an *N*-methyl group in place of an *N*-cyclo­propyl­methyl group (Amato *et al.*, 1990 ▸). Elucidation of the conformational profile of naltrexone is of fundamental importance in order to determine mol­ecular requirements for the specific binding affinities of this drug, particularly through the possible position of groups responsible for pharmacological action.

The most common pharmaceutical form of this compound is naltrexone hydro­chloride tablets. The introduction of new crystalline forms of an active pharmaceutical compound provides an opportunity to improve the performance characteristics of a pharmaceutical product. There is a need for new crystalline forms of naltrexone hydro­chloride (Nichols *et al.*, 2013 ▸) as well for new analytical methods of its unambiguous identification. This communication is a continuation of our work on analytical crystallography of opiate compounds (Gauchat & Naza­renko, 2017 ▸).
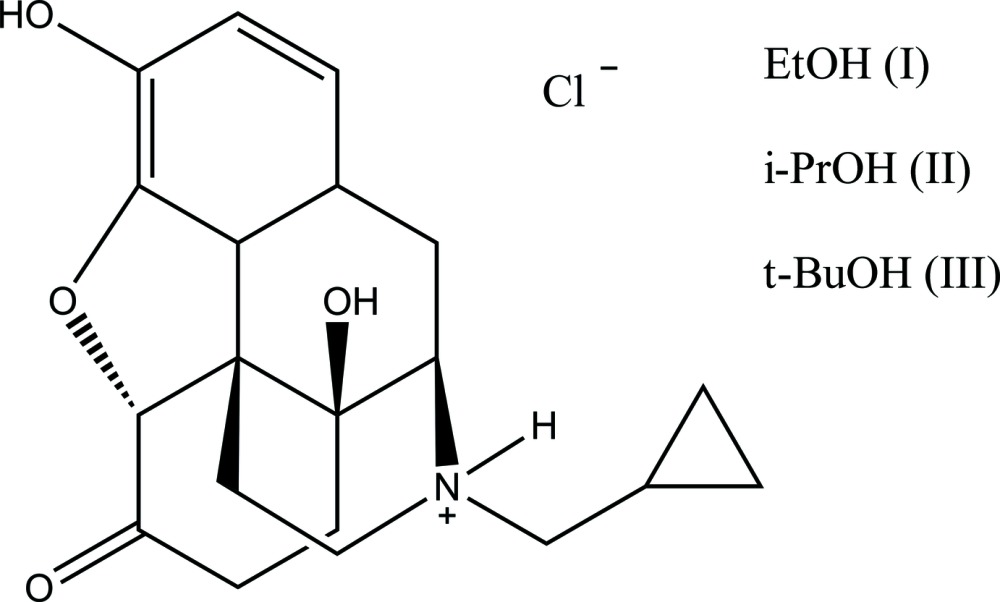


## Structural commentary   

**Figure 1:**
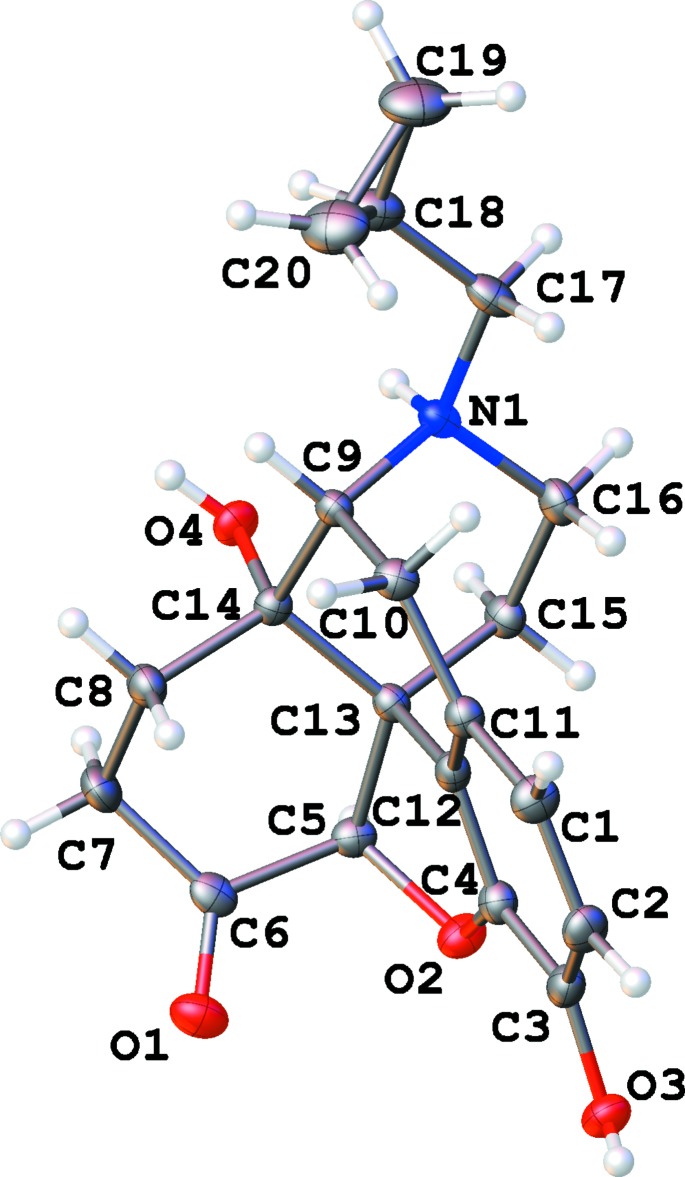
The numbering scheme of the naltrexone cation in the ethanol solvate structure (I), with 50% probability ellipsoids. All other naltrexone cations have the same numbering scheme (100 added to each atom number in a second naltrexone cation in structure III).

In all cases, inter­action with the alcohol mol­ecules does not affect the geometry of the methorphan ring system (Fig. 1 ▸), leaving the shape of the organic mol­ecule intact. The bond lengths and angles in the alcohol solvates are not far from expected values and are generally close to those reported for the hydrate structure (Ledain *et al.*, 1992 ▸).

There are four six-membered rings and a five-membered ring in a naltrexone mol­ecule. The aromatic ring is close to planar, with deviations less than 0.03 Å in all cases. The cyclo­hexadiene ring can be described as a half-chair shifted towards an envelope conformation: atoms C10, C11, C12 and C13 are adjacent to the aromatic ring and therefore almost planar while C9 and C14 deviate from this plane in opposite directions (see Table 1 ▸ for puckering parameters). A similar observation is true for the di­hydro­furane five-membered ring, which is almost inter­mediate between an envelope and a half-chair with C5 and C13 deviating from the mean plane in opposite directions.

The cyclo­hexa­none and piperidine rings both have chair conformations, with cyclo­hexa­none visibly shifted towards half-chair. These two rings are nearly coplanar. As a result, the naltrexone cation can be described as having two ring systems: a phenyl ring with adjacent ep­oxy and cyclo­hexadiene rings (tetra­hydro-2*H*-naphtho­[1,8-bc]furan system, atoms O2/C1–C4/ C9–C13) and cyclo­hexa­none plus piperidine rings (deca­hydro­isoquinolinium moiety, atoms N1/C5–C9/C13–C16). They are nearly perpendicular to each other, thus forming the well-established T-shape common to morphine, naloxene, and numerous similar mol­ecules (Darling *et al.*, 1982 ▸; Klein *et al.*, 1987 ▸; Gelbrich *et al.*, 2012 ▸). The angle between two mean planes is 83.9 (1)° for EtOH (I), 83.4 (1)° for *i*-PrOH (II) and 82.5 (1) and 84.3 (1)° for the two cations in *t*-BuOH (III) solvate.

**Figure 2:**
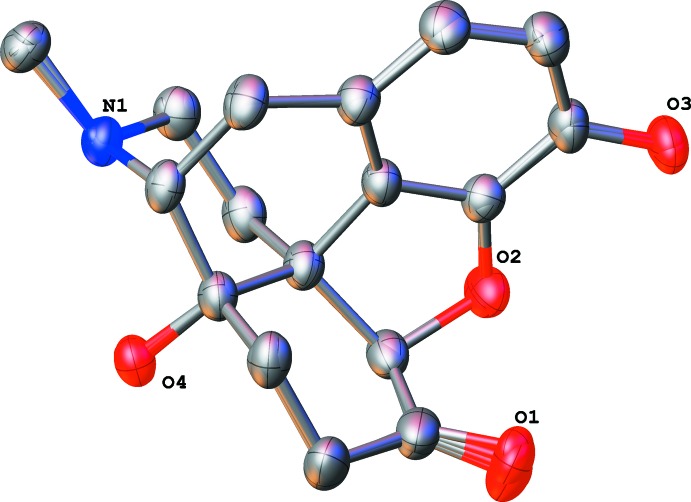
Overlay of all four naltrexone cations studied in this work with the cyclo­propyl group omitted.

What is responsible for switching from a potent opiate agonist (morphine and oxymorphone) to a potent competitive antagonist (naloxene and naltrexone)? It seems certain that changes in a relatively rigid oxymorphone cation are not liable. Overlay calculations show that all three naltrexone solvates fit the same shape (Fig. 2 ▸), with r.m.s. deviations being 0.09 (EtOH/*i*-PrOH), 0.06 and 0.11 Å (EtOH/*t*-BuOH). The same overlay with an oxymorphone cation (refcode BIZGAS) shows r.m.s. deviations of 0.10 to 0.13 Å and 0.13 Å for naloxene (refcode NALOXC02). It should be taken into account that, in these cases, the temperature of the experiment was different, which obviously increases the discrepancy. Even when we compare morphine (refcode EFASAH; Gelbrich *et al.*, 2012 ▸) and oxymorphone and morphine and naltrexone, the fit is almost identical: r.m.s. deviations of 0.36 and 0.35 Å, respectively, with larger discrepancies coming from obvious structural differences between the phenol group of morphine and a cyclo­hexa­none fragment in oxymorphone and naltrexone. The only flexible locations in the oxymorphone cation are oxygen O1 of the carbonyl group and the orientation of two hydroxyl groups (oxygen atoms O3 and O4), which all potentially form strong hydrogen bonds.

**Figure 3:**
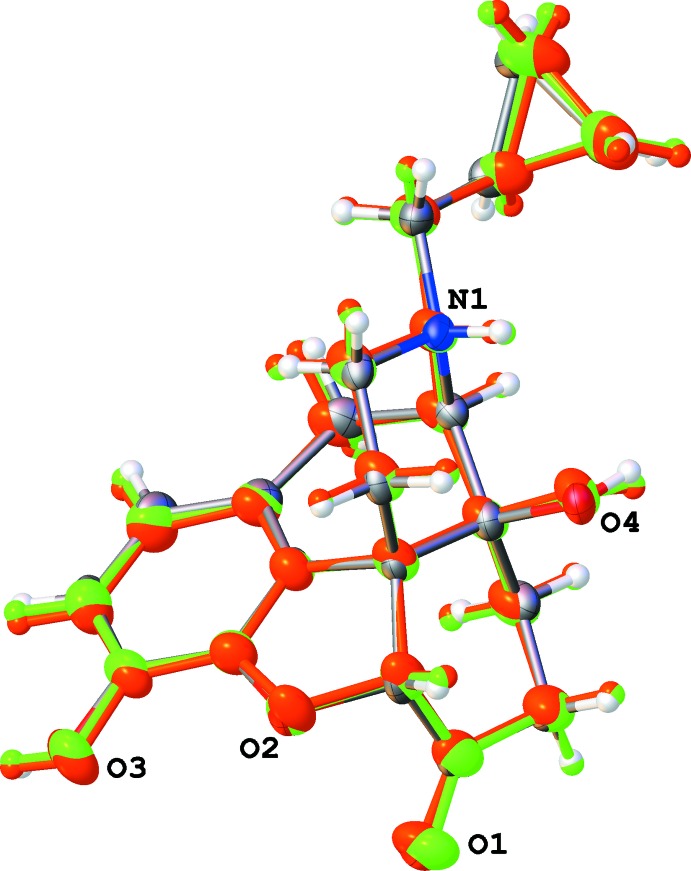
Overlay of both naltrexone cations of the *tert*-butanol solvate (III) (red and green) and of the propan-2-ol solvate (II) (usual color scheme). The orientation of the cyclo­propyl group is similar in all three cases.

**Figure 4:**
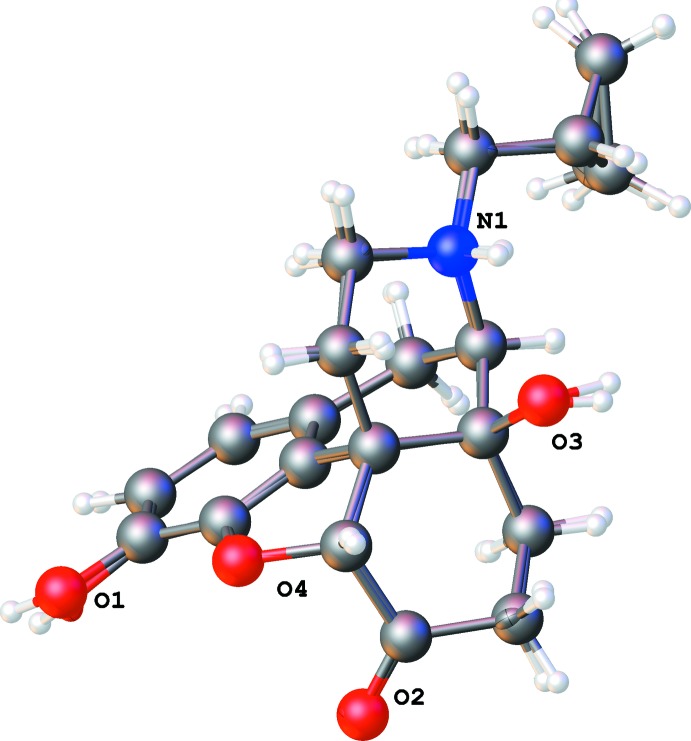
Overlay of the naltrexone cations of the ethanol solvate (I) and the tetra­hydrate (refcode PABCEA). The orientation of the cyclo­propyl group is similar in both cases.

**Figure 5:**
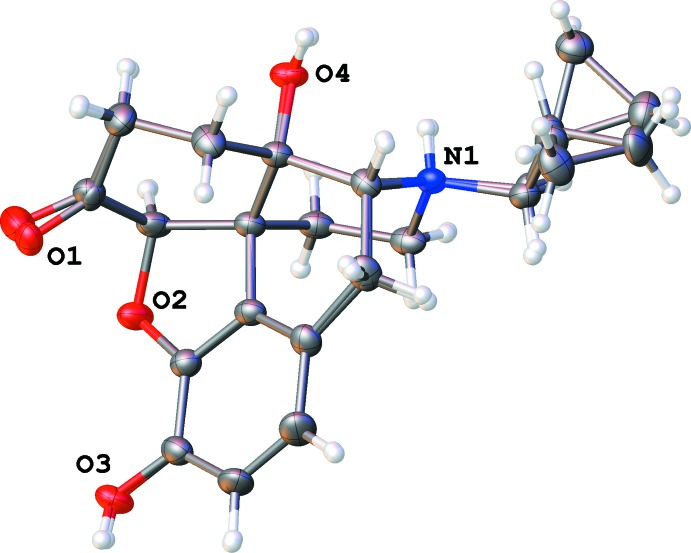
Overlay of the naltrexone cations of the ethanol solvate (I) and propanol solvate (II). The orientation of the cyclo­propyl group is visibly different.

Therefore, the simplest explanation of antagonist activity is the presence of a small ‘flat’ fragment attached to an *N*-methyl group: cyclo­propyl in naltrexone or vinyl in naloxene. The link between this small rigid fragment and the oxymorphone cation is flexible: as a result, we see different orientations of the cyclo­propane ring in various solvates of naltrexone. These orientations can be systemized in two groups. First, an overlay of the hydrate (refcode PABCEA) and the ethanol solvate (this work) shows very similar conformations for these two structures (Fig. 3 ▸). The orientation of the cyclo­propyl group in the *iso*-propanol and *tert*-butanol solvates is also almost the same (Fig. 4 ▸). However, these two groups significantly differ from each other (Fig. 5 ▸). The angle between the cyclo­propyl group plane and the mean plane of the cyclo­hexa­none and piperidine rings can serve as a qu­anti­tative measure of the methyl­cyclo­propyl fragment orientation. This angle is 36.1° (formate, H_2_O), 38.6° (H_2_O), 48.6° (EtOH), 71.7° (*i*-PrOH), 83.5° and 84.6°Å (*t*-BuOH); the first two values were calculated from Scheins *et al.* (2007 ▸) and Ledain *et al.* (1992 ▸). Thus, the conformation of the methyl­cyclo­propyl fragment is very sensitive to its environment.

## Supra­molecular features   

The way in which a solvate mol­ecule inter­acts with a naltrexone cation is different in all cases studied. Obviously, the strongest possible inter­action is a hydrogen bond associated with the hydroxyl group of the alcohol mol­ecule. However, naltrexone hydro­chloride is an ionic compound and electrostatic inter­action between a positively charged bulk cation and a chloride ion plays an essential role in crystal formation. Electrostatic potential data (Scheins *et al.*, 2007 ▸) show more or less uniform positive charge for most of the cation surface, with the obvious exception of the negatively charged oxygen atoms.

**Figure 6:**
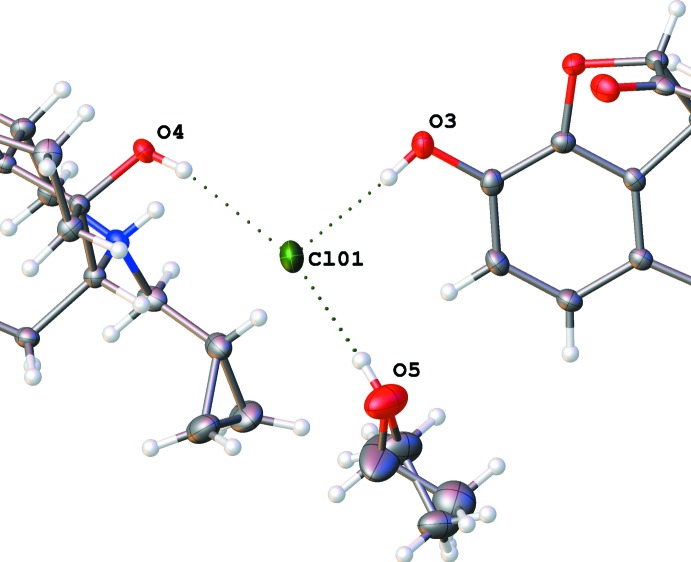
Hydrogen bonds around the chloride ion in the ethanol solvate (I).

**Figure 7:**
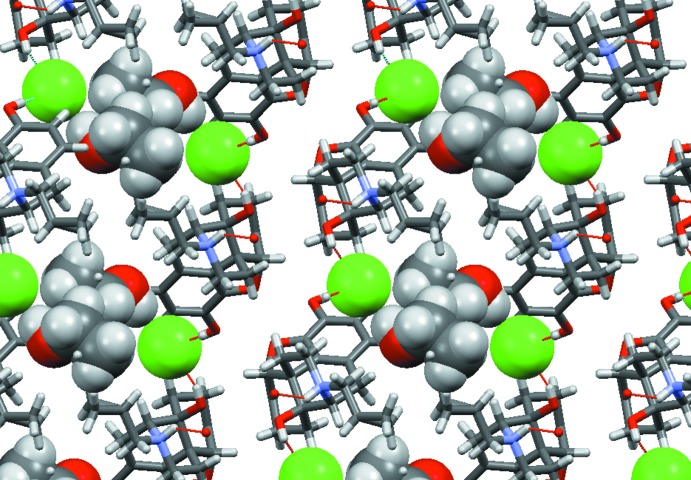
Packing diagram of the ion associates in the ethanol solvate (I), viewed along [010]. There is a visible gap between the bilayers. Chloride ions (green) and ethanol mol­ecules are highlighted.

In the ethanol solvate (I), the ethanol mol­ecule is disordered; however, both orientations show strong hydrogen bonds with the chloride anion and no direct inter­action with the naltrexone cation. The chloride ion is surrounded by hydroxyl groups belonging to two different cations (Fig. 6 ▸, Table 1 ▸). Inter­estingly, there is no hydrogen bond between the chloride ion and the formally positively charged protonated ammonium nitro­gen atom N1. Instead, there is a strong hydrogen bond between N1 and oxygen atom O1 of the carbonyl group belonging to another cation (Table 2 ▸). As a result, the naltrexone cations form infinite chains along the [010] direction. These chains are bound together *via* hydrogen bonds involving a chloride ion (Fig. 6 ▸), forming a layer in the (001) plane. Two pairs of these twin chains surround an infinite channel going along [010] axis containing the chloride ions and ethanol mol­ecules (Fig. 7 ▸), thus forming a double layer in the (001) plane. These layers are bound to each other only by weak van der Waals inter­actions, despite the overall positive charge of the cation chains. The shortest contact involves an O2 oxygen atom of one layer and an H15*A* hydrogen atom of another, and has an O—H separation of 2.60 (2) Å, which is above threshold of hydrogen bonding.

**Figure 8:**
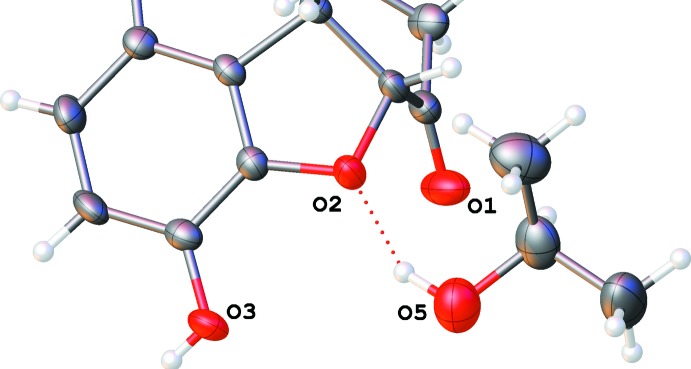
A dashed line indicates the O—H⋯O hydrogen bond connecting a propan-2-ol mol­ecule to an ether group of the naltrexone cation in (II). The minor component of the disordered propanol mol­ecule is omitted for clarity.

**Figure 9:**
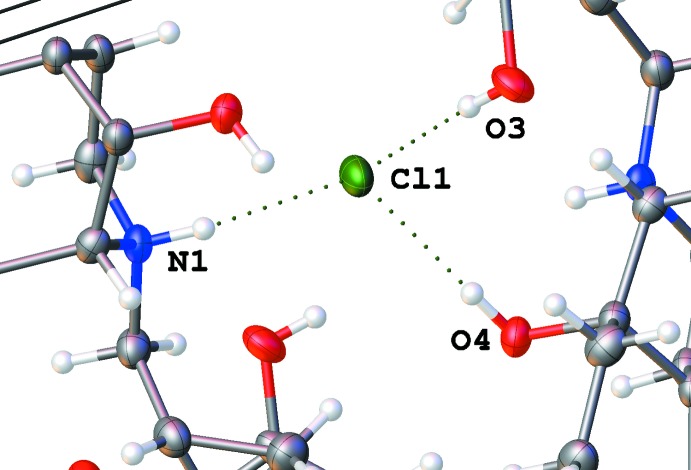
N—H⋯Cl and O—H⋯Cl hydrogen bonds around the chloride ion in the propan-2-ol solvate (II). Note that three different cations are connected.

**Figure 10:**
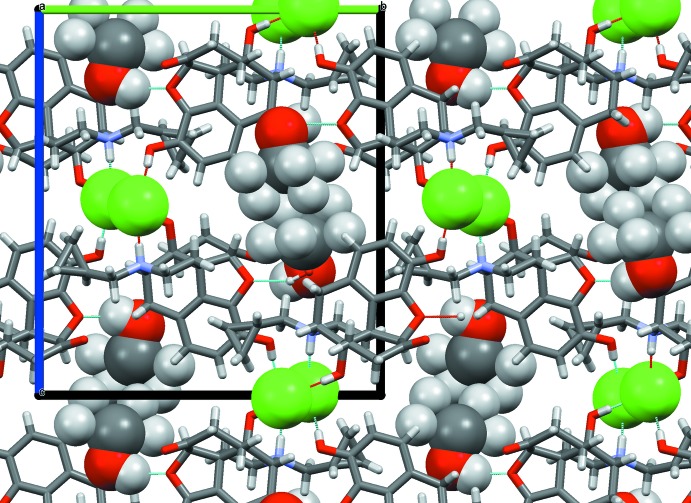
Packing diagram of the naltrexone ion associates in the propan-2-ol solvate (II), viewed along [100]. The chloride ions (green) and solvent mol­ecules are highlighted.

In the propan-2-ol solvate (II), the alcohol mol­ecule is also partially disordered. Both orientations make hydrogen bonds with ether oxygen atom, O2, of the di­hydro­furan ring (Fig. 8 ▸). In this structure, a chloride ion is surrounded by two hydroxyl groups and the protonated nitro­gen atom N1, all belonging to different naltrexone cations (Fig. 9 ▸, Table 3 ▸). These inter­actions result in a three-dimensional network, which has solvent-filled infinite channels oriented along the [100] direction (Fig. 10 ▸).

**Figure 11:**
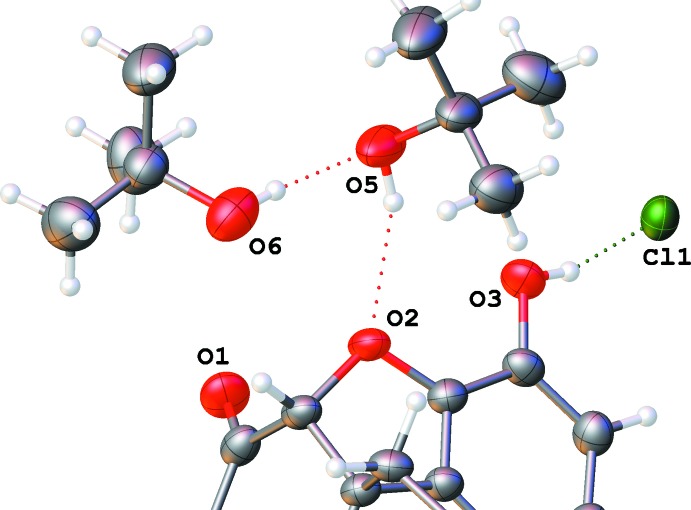
O—H⋯O hydrogen bonds connecting the *tert*-butanol mol­ecules in (III) to each other and to the ether group of a naltrexone cation.

**Figure 12:**
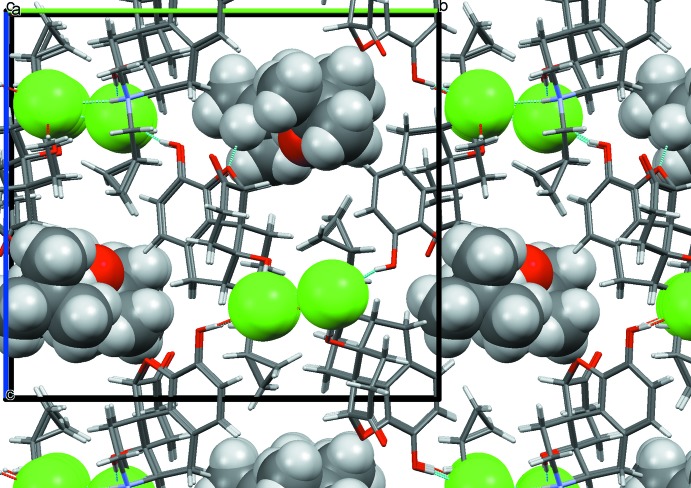
Packing diagram of the naltrexone ion associates in the 2-methyl­propan-2-ol (*tert*-butanol) solvate (III), viewed along [100]. The chloride ions (green) and solvent mol­ecules are highlighted.

In the 2-methyl­propan-2-ol (*tert*-butanol) solvate (III), two *tert*-butanol mol­ecules are connected *via* inter­molecular hydrogen bonds; one of them makes a hydrogen bond to oxygen atom O2 (Fig. 11 ▸) of the naltrexone cation. The same hydroxyl group is located close to another oxygen atom, O3, but the H5*A*⋯O3 separation (2.774 Å) is too long to be considered a real hydrogen bond. Another naltrexone cation in the same structure does not make direct hydrogen bonds to any solvate mol­ecule. Similar to the propanol solvate, both crystallographically independent chloride ions are surrounded by two hydroxyl groups and protonated nitro­gen atoms N1 and N101, all belonging to different naltrexone cations (Table 4 ▸). Again, the resulting three-dimensional network forms solvent-filled channels along the [100] direction (Fig. 12 ▸). Contrary to the ethanol solvate, in the propanol and *tert*-butanol solvates, sequences of chloride ions occupy locations which are separate from the solvent-filled channels.

In the tetra­hydrate (refcode PABCEA; Ledain *et al.*, 1992 ▸) and formate hydrate (refcode YIGREM; Scheins *et al.*, 2007 ▸), naltrexone cations form a chain *via* the protonated nitro­gen atom and an oxygen atom of a carbonyl group, similar to what we see in the ethanol solvate. Water mol­ecules and chloride ions also occupy a channel, this time along [001]. However, contrary to the ethanol solvate, the tetra­hydrate structure does not exhibit a layered layout.

It is worth mentioning that in the ethanol solvate of oxymorphone hydro­chloride (Darling *et al.*, 1982 ▸), the ethanol mol­ecule makes a weak hydrogen bond with the phenolic hy­droxy group (atom O3 in our numbering scheme).

A plausible assumption is that inter­action with an alcohol solvate mol­ecule (or absence of it) does not affect significantly the structure of the naltrexone cation. Obviously, the presence of a strong hydrogen bond at the cyclo­hexa­none carbonyl oxygen atom O1 (*e.g*., hydrate and ethanol solvate) is important; this affects the geometry of the cyclo­hexa­none moiety and, possibly, the orientation of the methyl­cyclo­propyl residue. Another significant factor is the size of a solvent-filled void. An increase of available space around the cyclo­propyl­methyl group may allow it to adopt a more favorable conformation.

## Database survey   

There are three reported naltrexone structures deposited in the Cambridge Structural Database (CSD Version 5.37; Groom *et al.*, 2016 ▸). Of these, two report the structures of the chloride salt at room temperature (refcodes XINSAP and PABCEA), one of which (Sugimoto *et al.*, 2007 ▸) is a powder structure of its anhydrous salt and the other (Ledain *et al.*, 1992 ▸) a single-crystal investigation of tetra­hydrate. A high-quality charge-density investigation of the neutral naltrexone mol­ecule and protonated naltrexone formate (refcodes YIGRAI and YIGREM; Scheins *et al.*, 2007 ▸) was performed at 100 K. A room-temperature structure of naltrexone malonate (refcode JEXRAF; Amato *et al.*, 1990 ▸) is also known. The existence of various solvates of naltrexone chloride was reported from powder data (Nichols *et al.*, 2013 ▸); however, no structural results were provided.

The crystal structure of oxymorphone hydro­chloride monohydrate ethanol solvate (refcode BIZGAS) is also known (Darling *et al.*, 1982 ▸). The experimental electron-density distribution of naloxone hydro­chloride dihydrate (refcode NALOXC02), another similar potent opiate antagonist, was described by Klein *et al.* (1987 ▸).

## Synthesis and crystallization   

Naltrexone hydro­chloride (INTAS Ltd, India) was obtained as a mixture with lactose. The target compound was extracted from its starting form by recrystallization in ethanol, *iso*-propanol, and *tert*-butanol. FTIR and Raman spectra of purified samples were consistent with database data for naltrexone hydro­chloride. A GC–MS study showed one single peak on the chromatogram with *m*/*z*: 341(*M* ^+^), 300 (*M* − C_3_H_5_), 286 (*M* − C_4_H_7_). A portion of the extracted naltrexone was then derivatized using penta­fluoro­propionic anhydride (PFPA), resulting in a corresponding di­penta­fluoro­propionate (*m*/*z*): 633 (*M*), 592 (*M* − C_3_H_5_), 486 (*M* − C_3_F_5_O). This is consistent with the existence of two hydroxyl groups in the naltrexone mol­ecule and confirms the correct chemical formula.

Nevertheless, diffractograms obtained from the crystallized material were all different from each other and from known naltrexone hydro­chloride hydrate and naltrexone hydro­chloride crystals (Ledain *et al.*, 1992 ▸; Sugimoto *et al.*, 2007 ▸; Nichols *et al.*, 2013 ▸). The quality of some of the solvate crystals was sufficient for single crystal investigation. Herein we report the results obtained.

## Refinement   

Crystal data, data collection and structure refinement details are summarized in Table 5 ▸.

In the ethanol solvate (I), the solvent mol­ecules are disordered with occupancies being approximately in a 2:1 ratio [0.66 (3):0.34 (3)]. Rigid body restrains (RIGU) were applied during refinement. In the propanol solvate (II), the occupancy of the minor component of a disordered solvent mol­ecule is only 0.178 (9), which required additional constraints (EXYZ and EADP) on the position of the hy­droxy group atoms. The *tert*-butanol solvate structure (III) was refined as a two-component twin (twin matrix: −1.000 0.000 0.000 −0.001 −1.000 0.000 0.164 0.000 1.000). There is visible flexibility in positions of the methyl groups of the tertiary *tert*-butanol mol­ecules, which results in larger displacement parameters and could be potentially treated as disorder. However, we do not see the need for additional over-complication of the refinement procedure.

Hydrogen atoms of the hydroxyl groups were refined with riding coordinates and stretchable bonds. Hydrogen atoms of the protonated amine were refined isotropically or with riding coordinates and stretchable bonds, with *U*_iso_ = 1.2*U*_iso_(N) in all cases. All other hydrogen atoms were refined with riding coordinates, with *U*_iso_ = 1.5*U*_iso_(C) for methyl groups and *U*_iso_ = 1.2*U*_iso_(C) for all others.

## Supplementary Material

Crystal structure: contains datablock(s) I, II, III. DOI: 10.1107/S205698901700843X/jj2196sup1.cif

Structure factors: contains datablock(s) I. DOI: 10.1107/S205698901700843X/jj2196Isup6.hkl

Structure factors: contains datablock(s) II. DOI: 10.1107/S205698901700843X/jj2196IIsup7.hkl

Structure factors: contains datablock(s) III. DOI: 10.1107/S205698901700843X/jj2196IIIsup8.hkl

CCDC references: 1554631, 1554630, 1554629

Additional supporting information:  crystallographic information; 3D view; checkCIF report

## Figures and Tables

**Table 1:** Ring puckering analysis (Å, °) of five- and six-membered rings Ring *A* di­hydro­furan (atoms O2/C4/C12/C13/C5), ring *B* piperidine (atoms N1/C9/C14/C13/C15/C16), ring *C* cyclo­hexa­none (atoms C5/C6/C7/C8/C13/C14) and ring *D* cyclo­hexadiene (atoms C9/C10/C11/C12/C13/C14).

Ring	parameter	(I)	(II)	(III) cation 1	(III) cation 2
*A*	*Q*	0.341 (2)	0.340 (3)	0.313 (3)	0.341 (3)
	φ	314.5 (4)	314.3 (5)	310.6 (5)	314.4 (5)
*B*	*Q*	0.637 (2)	0.624 (3)	0.637 (3)	0.636 (3)
	θ	11.28 (18)	10.9 (3)	9.3 (3)	9.6 (3)
	φ	101.0 (9)	110.8 (14)	102.1 (15)	97.6 (15)
*C*	*Q*	0.546 (3)	0.509 (3)	0.509 (3)	0.516 (3)
	θ	157.3 (2)	157.7 (3)	155.8 (3)	158.5 (3)
	φ	322.5 (7)	343.9 (10)	349.1 (9)	340.4 (10)
*D*	*Q*	0.495 (2)	0.502 (3)	0.499 (3)	0.508 (3)
	θ	131.6 (2)	134.1 (3)	134.2 (3)	132.1 (3)
	φ	121.2 (3)	122.7 (5)	123.7 (5)	122.4 (4)

**Table 2:** Hydrogen-bond geometry (Å, °) for (I)

*D*—H⋯*A*	*D*—H	H⋯*A*	*D*⋯*A*	*D*—H⋯*A*
N1—H1⋯O1^i^	0.85 (3)	2.23 (2)	2.870 (2)	133 (2)
O3—H3⋯Cl01	0.80 (2)	2.23 (2)	3.0297 (17)	171 (2)
O4—H4⋯Cl01^ii^	0.81 (3)	2.36 (3)	3.1279 (17)	159 (3)
O5—H5*A*⋯Cl01	0.84	2.33	3.160 (2)	169

Symmetry codes: (i) 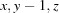; (ii) 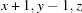.

**Table 3:** Hydrogen-bond geometry (Å, °) for (II)

*D*—H⋯*A*	*D*—H	H⋯*A*	*D*⋯*A*	*D*—H⋯*A*
N1—H1⋯Cl1^i^	0.87 (3)	2.34 (3)	3.102 (3)	146 (1)
O3—H3⋯Cl1^ii^	0.75 (4)	2.32 (4)	3.066 (3)	169 (4)
O4—H4⋯Cl1	0.85 (4)	2.21 (4)	3.054 (2)	177 (3)
O5—H5*A*⋯O2	1.00 (6)	2.00 (5)	2.921 (4)	154 (6)

Symmetry codes: (i) 
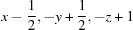
; (ii) 
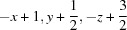
.

**Table 4:** Hydrogen-bond geometry (Å, °) for (III)

*D*—H⋯*A*	*D*—H	H⋯*A*	*D*⋯*A*	*D*—H⋯*A*
N1—H1⋯Cl1^i^	0.99 (3)	2.43 (3)	3.245 (3)	140 (3)
O3—H3⋯Cl1	0.84 (4)	2.20 (3)	2.999 (2)	162 (2)
O4—H4⋯Cl2^ii^	0.86 (3)	2.21 (3)	3.063 (2)	175 (1)
O5—H5*A*⋯O2	0.87 (5)	2.09 (3)	2.902 (3)	154 (4)
O6—H6⋯O5	0.84	2.03	2.867 (4)	174
N101—H101⋯Cl2^iii^	0.84 (2)	2.57 (2)	3.239 (3)	138 (2)
O103—H103⋯Cl2	0.87 (4)	2.15 (3)	3.002 (2)	164 (3)
O104—H104⋯Cl1	0.87 (3)	2.16 (3)	3.026 (2)	175 (1)

Symmetry codes: (i) 
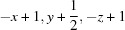
; (ii) 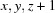; (iii) 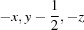.

**Table 5:** Experimental details

	(I)	(II)	(III)
Crystal data			
Chemical formula	C_20_H_24_NO_4_^+^·Cl^−^·C_2_H_6_O	C_20_H_24_NO_4_^+^·Cl^−^·C_3_H_8_O	C_20_H_24_NO_4_^+^·Cl^−^C_4_H_10_O
*M*_r_	423.92	437.94	451.97
Crystal system, space group	Monoclinic, *P*2_1_	Orthorhombic, *P*2_1_2_1_2_1_	Monoclinic, *P*2_1_
Temperature (K)	173	173	173
*a*, *b*, *c* (Å)	8.6885 (7), 7.9478 (6), 15.3417 (10)	8.0297 (10), 15.5449 (17), 17.560 (4)	8.8487 (4), 17.3281 (9), 15.5702 (8)
α, β, γ (°)	90, 103.908 (2), 90	90, 90, 90	90, 92.702 (2), 90
*V* (Å^3^)	1028.35 (13)	2191.9 (6)	2384.7 (2)
*Z*	2	4	4
Radiation type	Mo *K*α	Mo *K*α	Cu *K*α
μ (mm^−1^)	0.22	0.21	1.70
Crystal size (mm)	0.56 × 0.13 × 0.06	0.2 × 0.16 × 0.15	0.26 × 0.22 × 0.20

Data collection			
Diffractometer	Bruker PHOTON-100 CMOS	Bruker PHOTON-100 CMOS	Bruker PHOTON-100 CMOS
Absorption correction	Multi-scan (*SADABS*; Bruker, 2015 ▸)	Multi-scan (*SADABS*; Bruker, 2015 ▸)	Multi-scan (*TWINABS*; Bruker, 2012 ▸)
*T*_min_, *T*_max_	0.891, 1.000	0.925, 0.986	
No. of measured, independent and observed [*I* > 2σ(*I*)] reflections	33845, 5866, 5008	48846, 5327, 4481	9642, 9642, 8820
*R*_int_	0.042	0.043	
(sin θ/λ)_max_ (Å^−1^)	0.700	0.665	0.625

Refinement			
*R*[*F* ^2^ > 2σ(*F* ^2^)], *wR*(*F* ^2^), *S*	0.038, 0.082, 1.03	0.045, 0.112, 1.09	0.033, 0.079, 1.04
No. of reflections	5866	5327	9642
No. of parameters	305	313	581
No. of restraints	10	16	1
H-atom treatment	H atoms treated by a mixture of independent and constrained refinement	H atoms treated by a mixture of independent and constrained refinement	H atoms treated by a mixture of independent and constrained refinement
Δρ_max_, Δρ_min_ (e Å^−3^)	0.32, −0.24	0.33, −0.34	0.20, −0.17
Absolute structure	Flack *x* determined using 2015 quotients [(*I* ^+^)−(*I* ^−^)]/[(*I* ^+^)+(*I* ^−^)] (Parsons et al., 2013 ▸)	Flack *x* determined using 1682 quotients [(*I* ^+^)−(*I* ^−^)]/[(*I* ^+^)+(*I* ^−^)] (Parsons et al., 2013 ▸)	Flack *x* determined using 3828 quotients [(*I* ^+^)−(*I* ^−^)]/[(*I* ^+^)+(*I* ^−^)] (Parsons et al., 2013 ▸)
Absolute structure parameter	−0.017 (18)	−0.028 (18)	−0.004 (5)

Computer programs: *APEX2* and *SAINT* (Bruker, 2013 ▸), *SHELXT* (Sheldrick, 2015*a* ▸), *SHELXL2014* (Sheldrick, 2015*b* ▸), *OLEX2* (Dolomanov *et al.*, 2009 ▸), *Mercury* (Macrae *et al.*, 2006 ▸) and *PLATON* (Spek, 2009 ▸).

## supplementary crystallographic information

### Crystal data

**Table:**

C_20_H_24_NO_4_^+^·Cl^−^C_4_H_10_O	*F*(000) = 968
*M**_r_* = 451.97	*D*_x_ = 1.259 Mg m^−^^3^
Monoclinic, *P*2_1_	Cu *K*α radiation, λ = 1.54178 Å
*a* = 8.8487 (4) Å	Cell parameters from 9282 reflections
*b* = 17.3281 (9) Å	θ = 3.8–72.0°
*c* = 15.5702 (8) Å	µ = 1.70 mm^−^^1^
β = 92.702 (2)°	*T* = 173 K
*V* = 2384.7 (2) Å^3^	Block, colourless
*Z* = 4	0.26 × 0.22 × 0.20 mm

### Data collection

**Table:**

Bruker PHOTON-100 CMOS diffractometer	9642 independent reflections
Radiation source: sealedtube	8820 reflections with *I* > 2σ(*I*)
φ and ω scans	θ_max_ = 74.6°, θ_min_ = 2.8°
Absorption correction: multi-scan (*TWINABS*; Bruker, 2012)	*h* = −11→11
	*k* = −21→21
9642 measured reflections	*l* = 0→19

### Refinement

**Table:**

Refinement on *F*^2^	Hydrogen site location: mixed
Least-squares matrix: full	H atoms treated by a mixture of independent and constrained refinement
*R*[*F*^2^ > 2σ(*F*^2^)] = 0.033	*w* = 1/[σ^2^(*F*_o_^2^) + (0.0369*P*)^2^ + 0.2646*P*] where *P* = (*F*_o_^2^ + 2*F*_c_^2^)/3
*wR*(*F*^2^) = 0.079	(Δ/σ)_max_ < 0.001
*S* = 1.04	Δρ_max_ = 0.20 e Å^−^^3^
9642 reflections	Δρ_min_ = −0.17 e Å^−^^3^
581 parameters	Absolute structure: Flack *x* determined using 3828 quotients [(*I*^+^)-(*I*^-^)]/[(*I*^+^)+(*I*^-^)] (Parsons et al., 2013)
1 restraint	Absolute structure parameter: −0.004 (5)
Primary atom site location: dual	

### Special details

**Table:**

Geometry. All esds (except the esd in the dihedral angle between two l.s. planes) are estimated using the full covariance matrix. The cell esds are taken into account individually in the estimation of esds in distances, angles and torsion angles; correlations between esds in cell parameters are only used when they are defined by crystal symmetry. An approximate (isotropic) treatment of cell esds is used for estimating esds involving l.s. planes.
Refinement. Refined as a 2-component twin.

### Fractional atomic coordinates and isotropic or equivalent isotropic displacement parameters (Å^2^)

**Table:**

	*x*	*y*	*z*	*U*_iso_*/*U*_eq_	
Cl1	0.66572 (8)	0.25519 (4)	0.26921 (4)	0.03945 (16)	
Cl2	−0.15527 (8)	0.59450 (4)	−0.23882 (4)	0.03721 (15)	
O101	0.4508 (3)	0.37330 (15)	−0.11407 (14)	0.0567 (6)	
O102	0.1647 (3)	0.32376 (12)	−0.09967 (11)	0.0398 (5)	
O103	−0.0224 (3)	0.44419 (12)	−0.17614 (12)	0.0409 (5)	
H103	−0.077 (5)	0.485 (2)	−0.1889 (6)	0.061*	
O104	0.3849 (2)	0.24407 (11)	0.15125 (12)	0.0363 (4)	
H104	0.466 (4)	0.2502 (3)	0.184 (2)	0.054*	
N101	0.1251 (3)	0.28034 (14)	0.22373 (13)	0.0325 (5)	
H101	0.182 (2)	0.2418 (16)	0.2306 (3)	0.039*	
C101	−0.0057 (4)	0.49220 (17)	0.05622 (18)	0.0392 (7)	
H10A	−0.0398	0.5315	0.0932	0.047*	
C102	−0.0447 (4)	0.49543 (17)	−0.03114 (18)	0.0388 (7)	
H102	−0.1084	0.5361	−0.0520	0.047*	
C103	0.0063 (3)	0.44113 (16)	−0.08976 (17)	0.0345 (6)	
C104	0.0958 (3)	0.38254 (16)	−0.05536 (16)	0.0340 (6)	
C105	0.2807 (4)	0.29429 (17)	−0.03854 (17)	0.0381 (7)	
H105	0.2992	0.2383	−0.0495	0.046*	
C106	0.4274 (4)	0.34060 (18)	−0.04789 (18)	0.0426 (7)	
C107	0.5333 (4)	0.3442 (2)	0.02958 (19)	0.0467 (8)	
H10B	0.6185	0.3793	0.0182	0.056*	
H10C	0.5753	0.2923	0.0421	0.056*	
C108	0.4500 (4)	0.37357 (18)	0.10733 (18)	0.0404 (7)	
H10D	0.5220	0.3783	0.1577	0.049*	
H10E	0.4067	0.4252	0.0946	0.049*	
C109	0.2282 (3)	0.34699 (16)	0.20183 (16)	0.0334 (6)	
H109	0.2988	0.3561	0.2529	0.040*	
C110	0.1418 (4)	0.42301 (17)	0.18302 (17)	0.0389 (7)	
H11A	0.0552	0.4259	0.2209	0.047*	
H11B	0.2099	0.4669	0.1978	0.047*	
C111	0.0831 (3)	0.43183 (16)	0.09051 (17)	0.0349 (6)	
C112	0.1276 (3)	0.37784 (16)	0.03185 (17)	0.0318 (6)	
C113	0.2135 (3)	0.30510 (15)	0.05051 (16)	0.0323 (6)	
C114	0.3243 (3)	0.31769 (16)	0.12767 (17)	0.0332 (6)	
C115	0.1007 (3)	0.24068 (16)	0.07105 (17)	0.0364 (6)	
H11C	0.0276	0.2337	0.0215	0.044*	
H11D	0.1561	0.1915	0.0803	0.044*	
C116	0.0155 (3)	0.26007 (18)	0.15079 (17)	0.0371 (6)	
H11E	−0.0534	0.3041	0.1385	0.045*	
H11F	−0.0464	0.2152	0.1670	0.045*	
C117	0.0460 (3)	0.28935 (18)	0.30721 (17)	0.0365 (6)	
H11G	−0.0190	0.3358	0.3037	0.044*	
H11H	−0.0200	0.2441	0.3153	0.044*	
C118	0.1548 (4)	0.29650 (17)	0.38299 (17)	0.0382 (6)	
H118	0.2136	0.3457	0.3873	0.046*	
C119	0.2336 (4)	0.22618 (18)	0.41855 (19)	0.0466 (8)	
H11I	0.2121	0.1763	0.3895	0.056*	
H11J	0.3386	0.2322	0.4424	0.056*	
C120	0.1099 (5)	0.2623 (2)	0.46651 (19)	0.0528 (9)	
H12A	0.1385	0.2907	0.5200	0.063*	
H12B	0.0120	0.2347	0.4671	0.063*	
O1	0.0294 (3)	0.47448 (14)	0.37422 (13)	0.0511 (6)	
O2	0.3248 (2)	0.51172 (11)	0.40399 (11)	0.0368 (4)	
O3	0.5194 (3)	0.39454 (13)	0.34376 (12)	0.0436 (5)	
H3	0.570 (5)	0.355 (2)	0.3345 (7)	0.065*	
O4	0.1098 (2)	0.60254 (11)	0.64191 (12)	0.0358 (4)	
H4	0.036 (4)	0.5974 (3)	0.675 (2)	0.054*	
N1	0.3739 (3)	0.56903 (14)	0.72755 (13)	0.0324 (5)	
H1	0.310 (4)	0.6157 (19)	0.731 (2)	0.039*	
C1	0.5082 (4)	0.35396 (17)	0.57788 (19)	0.0402 (7)	
H1A	0.5453	0.3171	0.6188	0.048*	
C2	0.5475 (3)	0.34791 (18)	0.49230 (19)	0.0403 (7)	
H2	0.6140	0.3078	0.4767	0.048*	
C3	0.4921 (3)	0.39885 (17)	0.42884 (17)	0.0355 (6)	
C4	0.3974 (3)	0.45702 (16)	0.45557 (17)	0.0326 (6)	
C5	0.2118 (3)	0.54665 (16)	0.45837 (16)	0.0345 (6)	
H5	0.1983	0.6025	0.4435	0.041*	
C6	0.0611 (4)	0.50333 (16)	0.44294 (18)	0.0369 (6)	
C7	−0.0406 (4)	0.49965 (19)	0.51721 (19)	0.0422 (7)	
H7A	−0.1257	0.4641	0.5030	0.051*	
H7B	−0.0834	0.5515	0.5273	0.051*	
C8	0.0453 (3)	0.47173 (17)	0.59927 (18)	0.0366 (6)	
H8A	−0.0252	0.4675	0.6466	0.044*	
H8B	0.0889	0.4200	0.5894	0.044*	
C9	0.2696 (3)	0.50184 (16)	0.70365 (16)	0.0331 (6)	
H9	0.2003	0.4946	0.7519	0.040*	
C10	0.3548 (4)	0.42536 (17)	0.69254 (17)	0.0381 (7)	
H10F	0.4407	0.4233	0.7355	0.046*	
H10G	0.2859	0.3821	0.7048	0.046*	
C11	0.4147 (3)	0.41385 (16)	0.60374 (17)	0.0349 (6)	
C12	0.3663 (3)	0.46504 (15)	0.54039 (16)	0.0310 (6)	
C13	0.2796 (3)	0.53846 (15)	0.55069 (16)	0.0310 (6)	
C14	0.1716 (3)	0.52858 (15)	0.62424 (16)	0.0314 (6)	
C15	0.3919 (3)	0.60404 (16)	0.57368 (16)	0.0343 (6)	
H15A	0.4629	0.6101	0.5269	0.041*	
H15B	0.3357	0.6531	0.5790	0.041*	
C16	0.4808 (3)	0.58751 (18)	0.65763 (16)	0.0360 (6)	
H16A	0.5498	0.5434	0.6498	0.043*	
H16B	0.5429	0.6331	0.6745	0.043*	
C17	0.4593 (3)	0.56109 (18)	0.81328 (17)	0.0376 (6)	
H17A	0.5250	0.5149	0.8120	0.045*	
H17B	0.5252	0.6067	0.8228	0.045*	
C18	0.3563 (4)	0.55394 (17)	0.88672 (17)	0.0389 (7)	
H18	0.2991	0.5044	0.8905	0.047*	
C19	0.4090 (4)	0.5899 (2)	0.97063 (18)	0.0524 (8)	
H19A	0.3863	0.5624	1.0242	0.063*	
H19B	0.5066	0.6180	0.9727	0.063*	
C20	0.2796 (4)	0.62445 (19)	0.9190 (2)	0.0472 (8)	
H20A	0.2972	0.6739	0.8892	0.057*	
H20B	0.1769	0.6183	0.9407	0.057*	
O5	0.4669 (3)	0.57652 (18)	0.25608 (17)	0.0659 (8)	
H5A	0.4432 (18)	0.545 (3)	0.297 (3)	0.099*	
C21	0.6265 (4)	0.5937 (3)	0.2645 (2)	0.0548 (9)	
C22	0.6449 (5)	0.6660 (3)	0.2117 (3)	0.0857 (16)	
H22A	0.5998	0.6579	0.1537	0.129*	
H22B	0.7527	0.6778	0.2082	0.129*	
H22C	0.5940	0.7091	0.2390	0.129*	
C23	0.7149 (5)	0.5272 (3)	0.2279 (3)	0.0768 (13)	
H23A	0.6925	0.4797	0.2589	0.115*	
H23B	0.8234	0.5382	0.2344	0.115*	
H23C	0.6858	0.5209	0.1668	0.115*	
C24	0.6731 (4)	0.6062 (3)	0.3577 (2)	0.0644 (10)	
H24A	0.6150	0.6491	0.3805	0.097*	
H24B	0.7813	0.6184	0.3630	0.097*	
H24C	0.6534	0.5592	0.3904	0.097*	
O6	0.2828 (3)	0.69659 (15)	0.32281 (17)	0.0634 (7)	
H6	0.3391	0.6639	0.3005	0.095*	
C31	0.1740 (4)	0.7253 (2)	0.2583 (2)	0.0490 (8)	
C32	0.0606 (6)	0.7709 (3)	0.3054 (3)	0.0751 (12)	
H32A	0.0103	0.7371	0.3457	0.113*	
H32B	−0.0149	0.7927	0.2642	0.113*	
H32C	0.1121	0.8128	0.3373	0.113*	
C33	0.2538 (5)	0.7753 (3)	0.1944 (3)	0.0766 (13)	
H33A	0.2990	0.8201	0.2242	0.115*	
H33B	0.1806	0.7929	0.1494	0.115*	
H33C	0.3334	0.7452	0.1683	0.115*	
C34	0.1000 (5)	0.6567 (3)	0.2120 (3)	0.0744 (12)	
H34A	0.1780	0.6253	0.1861	0.112*	
H34B	0.0281	0.6752	0.1668	0.112*	
H34C	0.0464	0.6253	0.2532	0.112*	

### Atomic displacement parameters (Å^2^)

**Table:**

	*U*^11^	*U*^22^	*U*^33^	*U*^12^	*U*^13^	*U*^23^
Cl1	0.0394 (4)	0.0395 (4)	0.0390 (4)	0.0071 (3)	−0.0031 (3)	−0.0032 (3)
Cl2	0.0407 (4)	0.0366 (3)	0.0348 (3)	0.0083 (3)	0.0064 (3)	0.0045 (3)
O101	0.0668 (16)	0.0700 (16)	0.0344 (12)	0.0128 (13)	0.0152 (11)	0.0156 (10)
O102	0.0554 (13)	0.0410 (11)	0.0228 (9)	0.0142 (10)	−0.0012 (9)	−0.0015 (7)
O103	0.0544 (14)	0.0419 (11)	0.0258 (9)	0.0102 (10)	−0.0034 (9)	0.0043 (8)
O104	0.0388 (11)	0.0355 (10)	0.0340 (10)	0.0020 (9)	−0.0028 (8)	0.0083 (8)
N101	0.0380 (13)	0.0331 (12)	0.0260 (11)	−0.0001 (10)	−0.0022 (10)	0.0003 (8)
C101	0.0500 (19)	0.0358 (15)	0.0323 (15)	0.0072 (13)	0.0061 (13)	−0.0032 (11)
C102	0.0448 (17)	0.0379 (15)	0.0338 (15)	0.0090 (13)	0.0024 (13)	0.0058 (11)
C103	0.0398 (16)	0.0374 (15)	0.0258 (13)	0.0011 (12)	−0.0022 (11)	0.0057 (11)
C104	0.0417 (16)	0.0355 (14)	0.0248 (13)	0.0023 (12)	0.0023 (11)	−0.0014 (10)
C105	0.0515 (18)	0.0364 (15)	0.0264 (13)	0.0147 (14)	0.0023 (12)	−0.0005 (11)
C106	0.054 (2)	0.0401 (16)	0.0348 (16)	0.0176 (15)	0.0113 (13)	0.0051 (12)
C107	0.0446 (18)	0.055 (2)	0.0412 (17)	0.0027 (16)	0.0067 (14)	0.0161 (14)
C108	0.0469 (18)	0.0418 (16)	0.0322 (15)	−0.0051 (14)	−0.0023 (13)	0.0093 (12)
C109	0.0437 (16)	0.0331 (14)	0.0230 (12)	−0.0057 (12)	−0.0020 (11)	0.0012 (10)
C110	0.0562 (19)	0.0344 (15)	0.0262 (13)	0.0010 (14)	0.0019 (13)	−0.0030 (11)
C111	0.0450 (17)	0.0334 (14)	0.0264 (13)	0.0020 (12)	0.0042 (11)	0.0009 (10)
C112	0.0381 (15)	0.0315 (14)	0.0256 (13)	0.0015 (12)	−0.0014 (11)	0.0012 (10)
C113	0.0423 (16)	0.0328 (14)	0.0217 (12)	0.0027 (12)	−0.0001 (11)	0.0003 (10)
C114	0.0402 (15)	0.0325 (14)	0.0266 (13)	0.0022 (12)	−0.0017 (11)	0.0049 (10)
C115	0.0477 (17)	0.0328 (14)	0.0279 (13)	−0.0011 (13)	−0.0067 (12)	−0.0015 (10)
C116	0.0393 (16)	0.0397 (15)	0.0317 (14)	−0.0052 (13)	−0.0054 (12)	−0.0015 (12)
C117	0.0394 (16)	0.0413 (15)	0.0290 (13)	−0.0001 (13)	0.0039 (12)	0.0002 (11)
C118	0.0473 (18)	0.0406 (16)	0.0269 (13)	−0.0029 (14)	0.0031 (12)	−0.0034 (11)
C119	0.060 (2)	0.0448 (17)	0.0339 (15)	0.0020 (15)	−0.0078 (14)	−0.0048 (12)
C120	0.076 (2)	0.056 (2)	0.0265 (14)	−0.0048 (19)	0.0075 (15)	−0.0012 (13)
O1	0.0558 (14)	0.0648 (15)	0.0321 (11)	0.0027 (12)	−0.0044 (10)	−0.0109 (10)
O2	0.0459 (12)	0.0403 (11)	0.0246 (9)	0.0059 (9)	0.0072 (8)	0.0036 (7)
O3	0.0497 (14)	0.0495 (13)	0.0322 (10)	0.0098 (10)	0.0088 (9)	−0.0066 (8)
O4	0.0384 (11)	0.0344 (10)	0.0351 (10)	0.0009 (9)	0.0069 (8)	−0.0064 (8)
N1	0.0375 (13)	0.0353 (12)	0.0247 (11)	−0.0029 (10)	0.0035 (10)	−0.0029 (9)
C1	0.0474 (18)	0.0362 (15)	0.0364 (15)	0.0039 (14)	−0.0036 (13)	0.0008 (12)
C2	0.0425 (17)	0.0375 (16)	0.0407 (16)	0.0060 (14)	0.0001 (13)	−0.0056 (12)
C3	0.0365 (15)	0.0402 (15)	0.0299 (14)	−0.0018 (13)	0.0037 (12)	−0.0067 (11)
C4	0.0359 (15)	0.0328 (14)	0.0292 (13)	−0.0015 (12)	0.0027 (11)	0.0015 (10)
C5	0.0442 (16)	0.0345 (14)	0.0251 (13)	0.0067 (12)	0.0051 (11)	0.0014 (10)
C6	0.0463 (17)	0.0342 (14)	0.0298 (14)	0.0073 (13)	−0.0034 (12)	−0.0025 (11)
C7	0.0406 (17)	0.0491 (18)	0.0368 (16)	−0.0016 (14)	0.0003 (13)	−0.0092 (13)
C8	0.0396 (16)	0.0412 (16)	0.0294 (14)	−0.0074 (13)	0.0052 (12)	−0.0048 (11)
C9	0.0398 (16)	0.0364 (15)	0.0236 (12)	−0.0063 (12)	0.0063 (11)	−0.0017 (10)
C10	0.0535 (19)	0.0359 (15)	0.0248 (13)	−0.0039 (14)	0.0010 (12)	0.0035 (11)
C11	0.0412 (16)	0.0341 (14)	0.0293 (14)	−0.0001 (13)	0.0012 (12)	0.0004 (10)
C12	0.0353 (14)	0.0322 (13)	0.0257 (13)	0.0005 (12)	0.0031 (11)	−0.0018 (10)
C13	0.0386 (15)	0.0292 (13)	0.0253 (13)	−0.0003 (11)	0.0034 (11)	0.0010 (10)
C14	0.0369 (15)	0.0311 (14)	0.0265 (12)	−0.0017 (12)	0.0050 (11)	−0.0020 (10)
C15	0.0410 (15)	0.0342 (14)	0.0284 (12)	−0.0055 (13)	0.0088 (11)	0.0002 (11)
C16	0.0378 (15)	0.0404 (15)	0.0303 (13)	−0.0067 (14)	0.0065 (11)	−0.0024 (12)
C17	0.0417 (17)	0.0415 (15)	0.0292 (14)	0.0017 (13)	−0.0018 (12)	−0.0008 (11)
C18	0.0467 (17)	0.0423 (16)	0.0273 (14)	−0.0024 (14)	−0.0011 (12)	0.0038 (11)
C19	0.070 (2)	0.059 (2)	0.0279 (14)	−0.001 (2)	0.0000 (14)	0.0004 (14)
C20	0.059 (2)	0.0463 (17)	0.0377 (16)	0.0036 (16)	0.0123 (15)	0.0032 (13)
O5	0.0436 (13)	0.096 (2)	0.0582 (15)	−0.0102 (14)	0.0012 (11)	0.0354 (14)
C21	0.0395 (17)	0.075 (2)	0.0502 (18)	−0.0067 (19)	0.0057 (14)	0.0212 (18)
C22	0.057 (3)	0.104 (4)	0.096 (3)	−0.011 (3)	0.003 (2)	0.053 (3)
C23	0.064 (3)	0.093 (3)	0.075 (3)	−0.017 (3)	0.019 (2)	−0.006 (2)
C24	0.056 (2)	0.082 (3)	0.055 (2)	−0.008 (2)	0.0016 (17)	0.0049 (19)
O6	0.0667 (18)	0.0617 (17)	0.0604 (16)	−0.0029 (13)	−0.0104 (13)	0.0094 (12)
C31	0.0449 (19)	0.0504 (19)	0.0513 (19)	−0.0031 (15)	−0.0002 (15)	0.0067 (14)
C32	0.086 (3)	0.073 (3)	0.067 (3)	0.013 (3)	0.013 (2)	−0.002 (2)
C33	0.062 (3)	0.085 (3)	0.082 (3)	−0.002 (2)	0.005 (2)	0.034 (2)
C34	0.058 (3)	0.072 (3)	0.092 (3)	0.000 (2)	−0.007 (2)	−0.020 (2)

### Geometric parameters (Å, º)

**Table:**

O101—C106	1.203 (4)	C2—H2	0.9500
O102—C104	1.387 (3)	C2—C3	1.396 (4)
O102—C105	1.459 (3)	C3—C4	1.387 (4)
O103—H103	0.87 (4)	C4—C12	1.369 (4)
O103—C103	1.358 (3)	C5—H5	1.0000
O104—H104	0.87 (4)	C5—C6	1.539 (4)
O104—C114	1.425 (3)	C5—C13	1.538 (4)
N101—H101	0.84 (3)	C6—C7	1.500 (4)
N101—C109	1.520 (4)	C7—H7A	0.9900
N101—C116	1.500 (3)	C7—H7B	0.9900
N101—C117	1.513 (3)	C7—C8	1.534 (4)
C101—H10A	0.9500	C8—H8A	0.9900
C101—C102	1.388 (4)	C8—H8B	0.9900
C101—C111	1.399 (4)	C8—C14	1.526 (4)
C102—H102	0.9500	C9—H9	1.0000
C102—C103	1.400 (4)	C9—C10	1.538 (4)
C103—C104	1.380 (4)	C9—C14	1.548 (4)
C104—C112	1.376 (4)	C10—H10F	0.9900
C105—H105	1.0000	C10—H10G	0.9900
C105—C106	1.539 (5)	C10—C11	1.517 (4)
C105—C113	1.545 (4)	C11—C12	1.380 (4)
C106—C107	1.494 (4)	C12—C13	1.498 (4)
C107—H10B	0.9900	C13—C14	1.535 (4)
C107—H10C	0.9900	C13—C15	1.541 (4)
C107—C108	1.533 (4)	C15—H15A	0.9900
C108—H10D	0.9900	C15—H15B	0.9900
C108—H10E	0.9900	C15—C16	1.521 (4)
C108—C114	1.520 (4)	C16—H16A	0.9900
C109—H109	1.0000	C16—H16B	0.9900
C109—C110	1.544 (4)	C17—H17A	0.9900
C109—C114	1.551 (4)	C17—H17B	0.9900
C110—H11A	0.9900	C17—C18	1.500 (4)
C110—H11B	0.9900	C18—H18	1.0000
C110—C111	1.516 (4)	C18—C19	1.502 (4)
C111—C112	1.378 (4)	C18—C20	1.496 (4)
C112—C113	1.493 (4)	C19—H19A	0.9900
C113—C114	1.530 (4)	C19—H19B	0.9900
C113—C115	1.541 (4)	C19—C20	1.493 (5)
C115—H11C	0.9900	C20—H20A	0.9900
C115—H11D	0.9900	C20—H20B	0.9900
C115—C116	1.520 (4)	O5—H5A	0.87 (5)
C116—H11E	0.9900	O5—C21	1.443 (4)
C116—H11F	0.9900	C21—C22	1.512 (6)
C117—H11G	0.9900	C21—C23	1.518 (6)
C117—H11H	0.9900	C21—C24	1.506 (5)
C117—C118	1.492 (4)	C22—H22A	0.9800
C118—H118	1.0000	C22—H22B	0.9800
C118—C119	1.497 (4)	C22—H22C	0.9800
C118—C120	1.500 (4)	C23—H23A	0.9800
C119—H11I	0.9900	C23—H23B	0.9800
C119—H11J	0.9900	C23—H23C	0.9800
C119—C120	1.491 (5)	C24—H24A	0.9800
C120—H12A	0.9900	C24—H24B	0.9800
C120—H12B	0.9900	C24—H24C	0.9800
O1—C6	1.202 (3)	O6—H6	0.8400
O2—C4	1.381 (3)	O6—C31	1.447 (4)
O2—C5	1.471 (3)	C31—C32	1.497 (5)
O3—H3	0.84 (4)	C31—C33	1.519 (5)
O3—C3	1.360 (3)	C31—C34	1.522 (5)
O4—H4	0.86 (4)	C32—H32A	0.9800
O4—C14	1.425 (3)	C32—H32B	0.9800
N1—H1	0.99 (3)	C32—H32C	0.9800
N1—C9	1.521 (4)	C33—H33A	0.9800
N1—C16	1.510 (3)	C33—H33B	0.9800
N1—C17	1.509 (3)	C33—H33C	0.9800
C1—H1A	0.9500	C34—H34A	0.9800
C1—C2	1.397 (4)	C34—H34B	0.9800
C1—C11	1.398 (4)	C34—H34C	0.9800
			
C104—O102—C105	104.2 (2)	C13—C5—H5	110.0
C103—O103—H103	109.5	C13—C5—C6	113.4 (2)
C114—O104—H104	109.5	O1—C6—C5	120.4 (3)
C109—N101—H101	105.7	O1—C6—C7	123.0 (3)
C116—N101—H101	105.7	C7—C6—C5	116.6 (2)
C116—N101—C109	112.4 (2)	C6—C7—H7A	109.4
C116—N101—C117	111.4 (2)	C6—C7—H7B	109.4
C117—N101—H101	105.7	C6—C7—C8	111.3 (2)
C117—N101—C109	114.9 (2)	H7A—C7—H7B	108.0
C102—C101—H10A	119.6	C8—C7—H7A	109.4
C102—C101—C111	120.9 (3)	C8—C7—H7B	109.4
C111—C101—H10A	119.6	C7—C8—H8A	109.7
C101—C102—H102	118.7	C7—C8—H8B	109.7
C101—C102—C103	122.6 (3)	H8A—C8—H8B	108.2
C103—C102—H102	118.7	C14—C8—C7	109.7 (2)
O103—C103—C102	124.6 (2)	C14—C8—H8A	109.7
O103—C103—C104	119.4 (3)	C14—C8—H8B	109.7
C104—C103—C102	116.0 (2)	N1—C9—H9	107.3
C103—C104—O102	127.2 (2)	N1—C9—C10	113.1 (2)
C112—C104—O102	111.9 (2)	N1—C9—C14	106.1 (2)
C112—C104—C103	120.9 (3)	C10—C9—H9	107.3
O102—C105—H105	110.1	C10—C9—C14	115.3 (2)
O102—C105—C106	109.1 (2)	C14—C9—H9	107.3
O102—C105—C113	104.7 (2)	C9—C10—H10F	108.7
C106—C105—H105	110.1	C9—C10—H10G	108.7
C106—C105—C113	112.5 (2)	H10F—C10—H10G	107.6
C113—C105—H105	110.1	C11—C10—C9	114.2 (2)
O101—C106—C105	120.5 (3)	C11—C10—H10F	108.7
O101—C106—C107	123.0 (3)	C11—C10—H10G	108.7
C107—C106—C105	116.5 (2)	C1—C11—C10	126.7 (3)
C106—C107—H10B	109.6	C12—C11—C1	116.1 (2)
C106—C107—H10C	109.6	C12—C11—C10	117.2 (3)
C106—C107—C108	110.2 (3)	C4—C12—C11	123.7 (3)
H10B—C107—H10C	108.1	C4—C12—C13	108.5 (2)
C108—C107—H10B	109.6	C11—C12—C13	127.8 (2)
C108—C107—H10C	109.6	C5—C13—C15	111.6 (2)
C107—C108—H10D	109.7	C12—C13—C5	99.1 (2)
C107—C108—H10E	109.7	C12—C13—C14	109.0 (2)
H10D—C108—H10E	108.2	C12—C13—C15	108.9 (2)
C114—C108—C107	109.7 (3)	C14—C13—C5	118.6 (2)
C114—C108—H10D	109.7	C14—C13—C15	109.1 (2)
C114—C108—H10E	109.7	O4—C14—C8	110.3 (2)
N101—C109—H109	107.5	O4—C14—C9	108.7 (2)
N101—C109—C110	113.2 (2)	O4—C14—C13	107.5 (2)
N101—C109—C114	105.9 (2)	C8—C14—C9	112.7 (2)
C110—C109—H109	107.5	C8—C14—C13	110.9 (2)
C110—C109—C114	114.9 (2)	C13—C14—C9	106.5 (2)
C114—C109—H109	107.5	C13—C15—H15A	109.4
C109—C110—H11A	108.7	C13—C15—H15B	109.4
C109—C110—H11B	108.7	H15A—C15—H15B	108.0
H11A—C110—H11B	107.6	C16—C15—C13	111.3 (2)
C111—C110—C109	114.3 (2)	C16—C15—H15A	109.4
C111—C110—H11A	108.7	C16—C15—H15B	109.4
C111—C110—H11B	108.7	N1—C16—C15	110.1 (2)
C101—C111—C110	126.8 (2)	N1—C16—H16A	109.6
C112—C111—C101	115.3 (2)	N1—C16—H16B	109.6
C112—C111—C110	117.7 (3)	C15—C16—H16A	109.6
C104—C112—C111	124.2 (3)	C15—C16—H16B	109.6
C104—C112—C113	108.7 (2)	H16A—C16—H16B	108.2
C111—C112—C113	127.1 (2)	N1—C17—H17A	109.1
C112—C113—C105	98.1 (2)	N1—C17—H17B	109.1
C112—C113—C114	109.5 (2)	H17A—C17—H17B	107.8
C112—C113—C115	108.9 (2)	C18—C17—N1	112.6 (2)
C114—C113—C105	117.6 (3)	C18—C17—H17A	109.1
C114—C113—C115	109.7 (2)	C18—C17—H17B	109.1
C115—C113—C105	112.2 (2)	C17—C18—H18	116.3
O104—C114—C108	110.7 (2)	C17—C18—C19	117.0 (3)
O104—C114—C109	108.3 (2)	C19—C18—H18	116.3
O104—C114—C113	107.1 (2)	C20—C18—C17	119.3 (3)
C108—C114—C109	112.3 (2)	C20—C18—H18	116.3
C108—C114—C113	112.1 (2)	C20—C18—C19	59.7 (2)
C113—C114—C109	106.2 (2)	C18—C19—H19A	117.8
C113—C115—H11C	109.4	C18—C19—H19B	117.8
C113—C115—H11D	109.4	H19A—C19—H19B	114.9
H11C—C115—H11D	108.0	C20—C19—C18	59.9 (2)
C116—C115—C113	111.1 (2)	C20—C19—H19A	117.8
C116—C115—H11C	109.4	C20—C19—H19B	117.8
C116—C115—H11D	109.4	C18—C20—H20A	117.7
N101—C116—C115	110.0 (2)	C18—C20—H20B	117.7
N101—C116—H11E	109.7	C19—C20—C18	60.3 (2)
N101—C116—H11F	109.7	C19—C20—H20A	117.7
C115—C116—H11E	109.7	C19—C20—H20B	117.7
C115—C116—H11F	109.7	H20A—C20—H20B	114.9
H11E—C116—H11F	108.2	C21—O5—H5A	109.5
N101—C117—H11G	109.1	O5—C21—C22	104.5 (3)
N101—C117—H11H	109.1	O5—C21—C23	109.2 (4)
H11G—C117—H11H	107.9	O5—C21—C24	109.8 (3)
C118—C117—N101	112.4 (2)	C22—C21—C23	110.7 (3)
C118—C117—H11G	109.1	C24—C21—C22	112.0 (4)
C118—C117—H11H	109.1	C24—C21—C23	110.4 (3)
C117—C118—H118	116.0	C21—C22—H22A	109.5
C117—C118—C119	119.8 (3)	C21—C22—H22B	109.5
C117—C118—C120	117.7 (3)	C21—C22—H22C	109.5
C119—C118—H118	116.0	H22A—C22—H22B	109.5
C119—C118—C120	59.7 (2)	H22A—C22—H22C	109.5
C120—C118—H118	116.0	H22B—C22—H22C	109.5
C118—C119—H11I	117.7	C21—C23—H23A	109.5
C118—C119—H11J	117.7	C21—C23—H23B	109.5
H11I—C119—H11J	114.9	C21—C23—H23C	109.5
C120—C119—C118	60.3 (2)	H23A—C23—H23B	109.5
C120—C119—H11I	117.7	H23A—C23—H23C	109.5
C120—C119—H11J	117.7	H23B—C23—H23C	109.5
C118—C120—H12A	117.8	C21—C24—H24A	109.5
C118—C120—H12B	117.8	C21—C24—H24B	109.5
C119—C120—C118	60.1 (2)	C21—C24—H24C	109.5
C119—C120—H12A	117.8	H24A—C24—H24B	109.5
C119—C120—H12B	117.8	H24A—C24—H24C	109.5
H12A—C120—H12B	114.9	H24B—C24—H24C	109.5
C4—O2—C5	104.94 (19)	C31—O6—H6	109.5
C3—O3—H3	109.5	O6—C31—C32	106.3 (3)
C14—O4—H4	109.5	O6—C31—C33	109.7 (3)
C9—N1—H1	107.4 (19)	O6—C31—C34	108.5 (3)
C16—N1—H1	104.0 (18)	C32—C31—C33	111.3 (3)
C16—N1—C9	112.3 (2)	C32—C31—C34	111.2 (3)
C17—N1—H1	106.9 (18)	C33—C31—C34	109.8 (4)
C17—N1—C9	114.8 (2)	C31—C32—H32A	109.5
C17—N1—C16	110.7 (2)	C31—C32—H32B	109.5
C2—C1—H1A	119.8	C31—C32—H32C	109.5
C2—C1—C11	120.5 (3)	H32A—C32—H32B	109.5
C11—C1—H1A	119.8	H32A—C32—H32C	109.5
C1—C2—H2	118.9	H32B—C32—H32C	109.5
C3—C2—C1	122.2 (3)	C31—C33—H33A	109.5
C3—C2—H2	118.9	C31—C33—H33B	109.5
O3—C3—C2	125.6 (3)	C31—C33—H33C	109.5
O3—C3—C4	118.0 (2)	H33A—C33—H33B	109.5
C4—C3—C2	116.3 (2)	H33A—C33—H33C	109.5
O2—C4—C3	126.5 (2)	H33B—C33—H33C	109.5
C12—C4—O2	112.5 (2)	C31—C34—H34A	109.5
C12—C4—C3	121.0 (3)	C31—C34—H34B	109.5
O2—C5—H5	110.0	C31—C34—H34C	109.5
O2—C5—C6	108.5 (2)	H34A—C34—H34B	109.5
O2—C5—C13	104.7 (2)	H34A—C34—H34C	109.5
C6—C5—H5	110.0	H34B—C34—H34C	109.5
			
O101—C106—C107—C108	124.1 (3)	O1—C6—C7—C8	129.6 (3)
O102—C104—C112—C111	175.1 (3)	O2—C4—C12—C11	174.5 (3)
O102—C104—C112—C113	−6.0 (3)	O2—C4—C12—C13	−7.9 (3)
O102—C105—C106—O101	−23.8 (4)	O2—C5—C6—O1	−30.2 (4)
O102—C105—C106—C107	154.2 (2)	O2—C5—C6—C7	149.9 (2)
O102—C105—C113—C112	−33.9 (3)	O2—C5—C13—C12	−31.2 (3)
O102—C105—C113—C114	−151.0 (2)	O2—C5—C13—C14	−148.7 (2)
O102—C105—C113—C115	80.3 (3)	O2—C5—C13—C15	83.3 (3)
O103—C103—C104—O102	0.6 (5)	O3—C3—C4—O2	0.8 (4)
O103—C103—C104—C112	179.8 (3)	O3—C3—C4—C12	−179.4 (3)
N101—C109—C110—C111	−86.3 (3)	N1—C9—C10—C11	−85.1 (3)
N101—C109—C114—O104	−48.8 (3)	N1—C9—C14—O4	−49.5 (3)
N101—C109—C114—C108	−171.4 (2)	N1—C9—C14—C8	−172.1 (2)
N101—C109—C114—C113	65.9 (2)	N1—C9—C14—C13	66.1 (3)
N101—C117—C118—C119	−76.9 (3)	N1—C17—C18—C19	−145.7 (3)
N101—C117—C118—C120	−146.1 (3)	N1—C17—C18—C20	−76.9 (4)
C101—C102—C103—O103	−176.5 (3)	C1—C2—C3—O3	−176.7 (3)
C101—C102—C103—C104	1.4 (5)	C1—C2—C3—C4	1.0 (4)
C101—C111—C112—C104	3.0 (5)	C1—C11—C12—C4	4.0 (4)
C101—C111—C112—C113	−175.6 (3)	C1—C11—C12—C13	−173.1 (3)
C102—C101—C111—C110	176.2 (3)	C2—C1—C11—C10	176.1 (3)
C102—C101—C111—C112	0.3 (5)	C2—C1—C11—C12	−0.2 (4)
C102—C103—C104—O102	−177.4 (3)	C2—C3—C4—O2	−177.2 (3)
C102—C103—C104—C112	1.8 (4)	C2—C3—C4—C12	2.6 (4)
C103—C104—C112—C111	−4.2 (5)	C3—C4—C12—C11	−5.4 (5)
C103—C104—C112—C113	174.6 (3)	C3—C4—C12—C13	172.3 (3)
C104—O102—C105—C106	−88.5 (3)	C4—O2—C5—C6	−93.1 (2)
C104—O102—C105—C113	32.1 (3)	C4—O2—C5—C13	28.3 (3)
C104—C112—C113—C105	24.4 (3)	C4—C12—C13—C5	24.1 (3)
C104—C112—C113—C114	147.5 (2)	C4—C12—C13—C14	148.6 (2)
C104—C112—C113—C115	−92.5 (3)	C4—C12—C13—C15	−92.5 (3)
C105—O102—C104—C103	162.4 (3)	C5—O2—C4—C3	166.5 (3)
C105—O102—C104—C112	−16.9 (3)	C5—O2—C4—C12	−13.3 (3)
C105—C106—C107—C108	−53.9 (3)	C5—C6—C7—C8	−50.4 (3)
C105—C113—C114—O104	−78.5 (3)	C5—C13—C14—O4	−77.4 (3)
C105—C113—C114—C108	43.1 (3)	C5—C13—C14—C8	43.3 (3)
C105—C113—C114—C109	166.0 (2)	C5—C13—C14—C9	166.2 (2)
C105—C113—C115—C116	−169.7 (2)	C5—C13—C15—C16	−169.0 (2)
C106—C105—C113—C112	84.4 (3)	C6—C5—C13—C12	86.9 (3)
C106—C105—C113—C114	−32.6 (3)	C6—C5—C13—C14	−30.6 (3)
C106—C105—C113—C115	−161.3 (2)	C6—C5—C13—C15	−158.6 (2)
C106—C107—C108—C114	62.1 (3)	C6—C7—C8—C14	62.0 (3)
C107—C108—C114—O104	62.9 (3)	C7—C8—C14—O4	61.7 (3)
C107—C108—C114—C109	−176.0 (2)	C7—C8—C14—C9	−176.6 (2)
C107—C108—C114—C113	−56.6 (3)	C7—C8—C14—C13	−57.4 (3)
C109—N101—C116—C115	57.1 (3)	C9—N1—C16—C15	56.5 (3)
C109—N101—C117—C118	−60.2 (3)	C9—N1—C17—C18	−59.8 (3)
C109—C110—C111—C101	176.2 (3)	C9—C10—C11—C1	173.3 (3)
C109—C110—C111—C112	−8.0 (4)	C9—C10—C11—C12	−10.4 (4)
C110—C109—C114—O104	−174.6 (2)	C10—C9—C14—O4	−175.5 (2)
C110—C109—C114—C108	62.9 (3)	C10—C9—C14—C8	61.9 (3)
C110—C109—C114—C113	−59.8 (3)	C10—C9—C14—C13	−59.9 (3)
C110—C111—C112—C104	−173.4 (3)	C10—C11—C12—C4	−172.6 (3)
C110—C111—C112—C113	8.0 (5)	C10—C11—C12—C13	10.2 (4)
C111—C101—C102—C103	−2.4 (5)	C11—C1—C2—C3	−2.2 (5)
C111—C112—C113—C105	−156.8 (3)	C11—C12—C13—C5	−158.4 (3)
C111—C112—C113—C114	−33.7 (4)	C11—C12—C13—C14	−33.9 (4)
C111—C112—C113—C115	86.3 (3)	C11—C12—C13—C15	85.0 (3)
C112—C113—C114—O104	170.8 (2)	C12—C13—C14—O4	170.5 (2)
C112—C113—C114—C108	−67.6 (3)	C12—C13—C14—C8	−68.8 (3)
C112—C113—C114—C109	55.3 (3)	C12—C13—C14—C9	54.1 (3)
C112—C113—C115—C116	−62.2 (3)	C12—C13—C15—C16	−60.7 (3)
C113—C105—C106—O101	−139.6 (3)	C13—C5—C6—O1	−146.1 (3)
C113—C105—C106—C107	38.5 (3)	C13—C5—C6—C7	34.0 (3)
C113—C115—C116—N101	−52.1 (3)	C13—C15—C16—N1	−52.5 (3)
C114—C109—C110—C111	35.6 (4)	C14—C9—C10—C11	37.2 (4)
C114—C113—C115—C116	57.6 (3)	C14—C13—C15—C16	58.1 (3)
C115—C113—C114—O104	51.4 (3)	C15—C13—C14—O4	51.8 (3)
C115—C113—C114—C108	173.0 (2)	C15—C13—C14—C8	172.4 (2)
C115—C113—C114—C109	−64.1 (3)	C15—C13—C14—C9	−64.6 (3)
C116—N101—C109—C110	62.6 (3)	C16—N1—C9—C10	63.9 (3)
C116—N101—C109—C114	−64.2 (3)	C16—N1—C9—C14	−63.4 (3)
C116—N101—C117—C118	170.5 (2)	C16—N1—C17—C18	171.8 (2)
C117—N101—C109—C110	−66.3 (3)	C17—N1—C9—C10	−63.6 (3)
C117—N101—C109—C114	166.9 (2)	C17—N1—C9—C14	169.1 (2)
C117—N101—C116—C115	−172.2 (2)	C17—N1—C16—C15	−173.8 (2)
C117—C118—C119—C120	−106.7 (3)	C17—C18—C19—C20	109.8 (3)
C117—C118—C120—C119	110.0 (3)	C17—C18—C20—C19	−106.0 (3)

### Hydrogen-bond geometry (Å, º)

**Table:**

*D*—H···*A*	*D*—H	H···*A*	*D*···*A*	*D*—H···*A*
N1—H1···Cl1^i^	0.99 (3)	2.43 (3)	3.245 (3)	140 (3)
O3—H3···Cl1	0.84 (4)	2.20 (3)	2.999 (2)	162 (2)
O4—H4···Cl2^ii^	0.86 (3)	2.21 (3)	3.063 (2)	175 (1)
O5—H5*A*···O2	0.87 (5)	2.09 (3)	2.902 (3)	154 (4)
O6—H6···O5	0.84	2.03	2.867 (4)	174
N101—H101···Cl2^iii^	0.84 (2)	2.57 (2)	3.239 (3)	138 (2)
O103—H103···Cl2	0.87 (4)	2.15 (3)	3.002 (2)	164 (3)
O104—H104···Cl1	0.87 (3)	2.16 (3)	3.026 (2)	175 (1)

Symmetry codes: (i) −*x*+1, *y*+1/2, −*z*+1; (ii) *x*, *y*, *z*+1; (iii) −*x*, *y*−1/2, −*z*.
